# Outpacing climate change: adaptation to heatwaves in Europe

**DOI:** 10.1007/s00484-025-02872-0

**Published:** 2025-02-19

**Authors:** Marcin Piotr Walkowiak, Karol Bandurski, Jarosław Walkowiak, Dariusz Walkowiak

**Affiliations:** 1 Department of Preventive Medicine, Poznan University of Medical Sciences, Święcickiego 6, 60-781 Poznań, Poland; 2 Institute of Environmental Engineering and Building Installations, Poznań University of Technology, Poznań, Poland; 3 Department of Pediatric Gastroenterology and Metabolic Diseases, Poznan University of Medical Sciences, Poznań, Poland; 4 Department of Organization and Management in Health Care, Poznan University of Medical Sciences, Poznań, Poland

**Keywords:** Heatwave, Adaptation, Climate change, Harvesting effect, Inequalities

## Abstract

Current predictions of climate change impacts rely on conservative assumptions about a lack of adaptation, projecting significantly increased heatwave mortality. However, long-term studies have shown a decline in actual heatwave deaths, raising questions about the underlying mechanisms. We combined Eurostat weekly mortality data (baseline extracted via Seasonal-Trend decomposition by Loess and smoothed through Principal Component Analysis dimension reduction and reconstruction) with economic indicators, Copernicus temperature data since 1950, and ENTSO-E electricity demand data. Panel regression analyzed mortality patterns during weeks with daily temperatures exceeding 22 °C for 2000–2022. During the analyzed period, Europe outpaced climate change, with the capacity to tolerate an additional 1 °C rise every 17.9 years [95% CI 15.3–22.7]. Extending the temperature indicators beyond the prior 3 years did not enhance predictive accuracy, suggesting swift adaptations and historical climate lacked any predictive value. Additionally, increasing economic output, likely driven by infrastructural improvements, especially greater affordability of air conditioning, enabled tolerating each additional 1 °C due to a per capita GDP increase of 19.7 thousand euros [95% CI 14.6–30.3]. Consistently, the increase in cooling energy demand was the strongest in eastern Europe. The findings shed light on the mechanisms driving the observed reduction in heatwave mortality despite the warming climate trend, offering a more plausible basis for extrapolation than assuming a lack of adaptation. The model emphasizes the role of long term economic growth and addressing energy poverty.

## Introduction

Given Europe's particularly rapid warming rate (European Environment Agency 2024) and the dominance of heatwaves as a source of extreme weather mortality (Jones et al. 2021), there appears to be a theoretical gap in assessing the long-term effects of heatwaves in the context of global warming. Studies on vulnerability to both low and high temperatures reveal regional differences, suggesting that populations are adapted to their local climates. However, even highly reputable climate change impact studies have traditionally assumed that these relationships will remain constant despite climate change (Gasparrini et al. 2017; Madaniyazi et al. 2024), leading to projections of significant increases in heatwave-related deaths. While some recent studies (Masselot et al. 2025) have started to explore adaptation scenarios, such considerations have not been a central focus in most projections. Notably, non-adaptation projections are difficult to reconcile with other studies – sometimes even coauthored by the same researchers – that assess the impact of heatwaves over recent decades. These works consistently find declining vulnerability and, in some cases, even a reduction in overall mortality despite a warming climate. This trend has been observed in the UK (Carson et al. 2006), Spain (Achebak et al. 2018; Huber et al. 2022), the Netherlands (Folkerts et al. 2020), Sweden (Oudin Åström et al. 2018), the USA (Sheridan and Dixon 2017), Australia (Coates et al. 2014; Osborne et al. 2024), East Asia (Chung et al. 2017) and in international studies (Wu et al. 2024). This raises questions about the underlying adaptation mechanisms, as modeling adaptations pose a well-known challenge (Vicedo-Cabrera et al. 2019; Cordiner et al. 2024).

The observed adaptations could be biological, with literature suggesting they operate relatively quickly. Studies on sports performance indicate that most adaptation occurs within the first two weeks, although a slightly longer period can still be beneficial (Tyler et al. 2016). Extreme cases, like pilgrims arriving in Mecca (Yezli et al. 2023) or migrants moving to countries 10 °C colder than their origin (Shor and Roelfs 2019), are typically required to demonstrate elevated mortality among population not adapted to local temperature ranges. Additionally, the 'harvesting effect' refers to a mechanism that, at the population level, may resemble a form of short-term adaptation. Heatwaves typically cause an initial spike in mortality, often followed by a period of below-trend mortality despite sustained high temperatures (Plavcová and Kyselý 2010; Qiao et al. 2015). This pattern arises because heatwaves disproportionately impact the most vulnerable individuals, accelerating their deaths by weeks or months. As a result, the remaining population temporarily exhibits reduced susceptibility to heatwave-related mortality.

Adaptations to specific temperature ranges can also be infrastructural. These can be passive, such as insulation (Schnieders et al. 2015) or high albedo roofs (Mastrapostoli et al. 2014). Alternatively, they can involve active systems like air conditioning, which are particularly important when high temperatures persist at night, preventing ambient cooling. Studies show a clear statistical relationship between prevalence of house air conditioning and a lower number of heatwave deaths (Sera et al. 2020; Chua et al. 2023). However, there is an exceptional spike of heatwave mortality during blackouts (Yamasaki et al. 2024). Some studies not only detected a clear reduction in mortality due to having air conditioning at home but also observed a subtle impact of the availability of air-conditioned public spaces (Eisenman et al. 2016). There is also a demonstrated impact on the reduction of hospitalization during heatwaves, which persisted even after correcting for socioeconomic status (Ostro et al. 2010), while hospitalized patients survival chances were improved in case of installation of air conditioning in hospital (Lenzer et al. 2020). As infrastructure develops over decades, there is no defined ceiling on the extent of adaptation, so its impact could not only be lagged but also extend beyond the initially observed level.

In many cases, adaptation occurs so naturally that it is taken for granted. Studies on retirement migrations show little consideration for the supposed challenge of adapting to a warmer climate, despite retirees overwhelmingly choosing warmer regions for relocation. Such migrations are even often explicitly justified on climate and health grounds (Breuer 2005). Alternatively, in studies analyzing health challenges, the primary focus is on financial, cultural, and linguistic barriers (Hall and Hardill 2016). Moreover, in some cases, infrastructural adaptations shield people so well from the local climate that they start to attenuate physiological adjustments, leading to people in air-conditioned environments becoming less adapted to higher temperatures (Yu et al. 2012; Pallubinsky et al. 2017).

The aim of this study is to analyze changes in resistance to high temperatures in Europe. The goal is to quantify and understand the process in a manner that permits a more plausible approximation of ongoing trends, facilitating modeling without the need to assume a fixed relationship. Moreover, analyzing predictors may shed light on the underlying mechanism, thus enabling the shaping of public policy.

## Methods

### Data source

Our primary analysis covered 2000–2022, constrained primarily by the availability of economic data. The dataset comprises mortality figures for NUTS1 regions (Nomenclature of Territorial Units for Statistics, level 1 – effectively large provinces or smaller countries) reported by ISO-weeks (standardized Monday-to-Sunday weeks following the International Organization for Standardization calendar). After excluding Great Britain and Albania due to mortality data quality concerns and lack of compatible economic data, the mortality dataset from Eurostat (Eurostat 2024) included 95 million deaths and extended through early 2024 for fitting mortality cycles. From the same database, we derived regional gross domestic product per inhabitant in purchasing power standard, adjusted to 2020 prices. Data on daily mean temperature were obtained from the Copernicus Climate Data Store, provided as "E-OBS daily gridded meteorological data for Europe from 1950 to the present," with a resolution of 0.25° × 0.25°(Copernicus 2024). Information on country-level hourly electricity consumption, used as a proxy variable for estimating air conditioning use, was derived from the European Network of Transmission System Operators for Electricity for 2006–2023. Selected regions were attributed to the Köppen-Geiger climate classification following the results of Beck et al. (Beck et al. 2018).

### Calculation

While other works have assumed a fixed regional relationship in their approximations of the mortality temperature relationship, we were concerned this would oversimplify the annual cycle. During the warm season there is a reduction in respiratory infections, leading to a steady decline in mortality. The longer the warm period, the deeper the interruption, and what may be only mildly noticeable in continental climates leads to significant cycle asymmetry in warm Mediterranean regions (Falagas et al. 2009; Walkowiak and Walkowiak 2024). Failing to adjust for the shifting baseline would overestimate the impact of heatwaves in early summer while underestimating their impact in late summer.

To correct for this factor, we used the Seasonal and Trend decomposition using the Loess (STL) model to estimate the annual cycle for log-transformed data, assuming a fixed cycle. Although STL is robust to outliers, it can produce improbable fluctuations when it independently estimates subsequent cyclical components. This is especially true if extreme values repeatedly occur in the same part of the cycle. To mitigate this, we preprocessed the data by replacing any spike—defined as a value higher than both the two preceding and the two succeeding observations—with the lowest of these values.

As regional estimations are highly susceptible to random noise, we again removed spikes and subsequently applied a Gaussian filter to smooth them. These cycles were then subjected to Principal Component Analysis with Singular Value Decomposition (PCA) into three dimensions, followed by inverse transformation to obtain estimates in the original feature space. After reversing the log transformation, this was used to calculate weekly mortality deviation from a seasonally adjusted trend value expressed as a share of the unadjusted trend value.

Temperature data from Copernicus was matched with their respective NUTS regions/countries. Temperatures from all grid points matching a particular area are averaged. When estimating weekly mortality, the highest daily temperature of each week is selected for each grid point in order to detect relatively short heatwaves.

To estimate the relationship between high temperature and mortality, we calculate the deviation from the mortality trend for weeks with a maximal average daily temperature of at least 22 °C. While this threshold may appear low, it aligns with research showing remarkably low optimal temperatures in Scandinavia, with studies indicating mortality minimums around 20 °C (Gasparrini et al. 2015) or some older suggesting even 11–12 °C (Rocklöv and Forsberg 2008). Our analysis includes only regions with at least 20 such weeks in their dataset. Sensitivity analyses with higher temperature thresholds, presented in Supplementary Materials, confirm that our main findings remain robust. The following explanatory variables are being tested:

- Highest daily temperature in particular week.
- Temperature change from the last week to adjust for the immediate harvesting effect (Plavcová and Kyselý 2010; Qiao et al. 2015).
- ISO week number, to adjust for longer term impact of harvesting effect and possible short term adaptation (Tyler et al. 2016).
- Year to reflect possible changes in high temperature sensitivity over time.
- Influenza deaths, representing excess mortality during weeks of the typical influenza season (W01-W15), are shown to reduce heatwave mortality by affecting the most vulnerable individuals a few months earlier. While the relationship between influenza deaths and reduced heatwave-related mortality in the following summer is well established (Stafoggia et al. 2009; Rocklöv et al. 2009; Ha et al. 2011; Qiao et al. 2015), this relationship may be oversimplified for COVID-19 deaths. COVID-19, caused by a more virulent strain, affected less vulnerable populations and was accompanied by a reverse harvesting effect: strict pandemic restrictions suppressed infectious diseases in general, allowing highly vulnerable individuals to survive who would have otherwise succumbed to non-specifically virulent infections (Walkowiak et al. 2023).
- Local economic development, represented by both the crude regional (NUTS1) gross domestic product per inhabitant in purchasing power standard adjusted to 2020 prices, and its log-transformed value to reflect diminishing returns of increasing prosperity.
- The 90th percentile of annual temperature from a region is typically indicative of elevated temperatures. This value is calculated for a range of 1 to 30 years to assess the adequacy of the time lag in explaining contemporary vulnerability, and for the period 1950–1969 to represent the fixed impact of the past climate before warming began to accelerate.

While observations from the same region cannot be considered independent, favoring panel regression, the Hausman test will be used to determine whether the random effects model might be more suitable. Additionally, the pFtest will verify the suitability of basic linear regression as included variables may reflect a significant share of the regional differences. The relationships between coefficients were estimated using bootstrapping with 10,000 iterations and a 95% confidence interval. To verify the plausibility of the increasing role of air conditioning as an adaptation to high temperatures per capita electricity consumption on working days are being plotted against temperature and modeled using Locally Estimated Scatterplot Smoothing (LOESS).

Given the limitations encountered in Python, the Hausman test and pFtest were carried out using R version 4.1.2 with plm_2.6–4, with all other tasks completed in Python 3.10. Libraries netCDF4 1.6.4 and geopandas 0.13.2 were used for extracting temperature data. Models were calculated with statsmodels 1.13.5, while matplotlib 3.7.1 was used for data visualization.

## Results

**Fig. 1:**
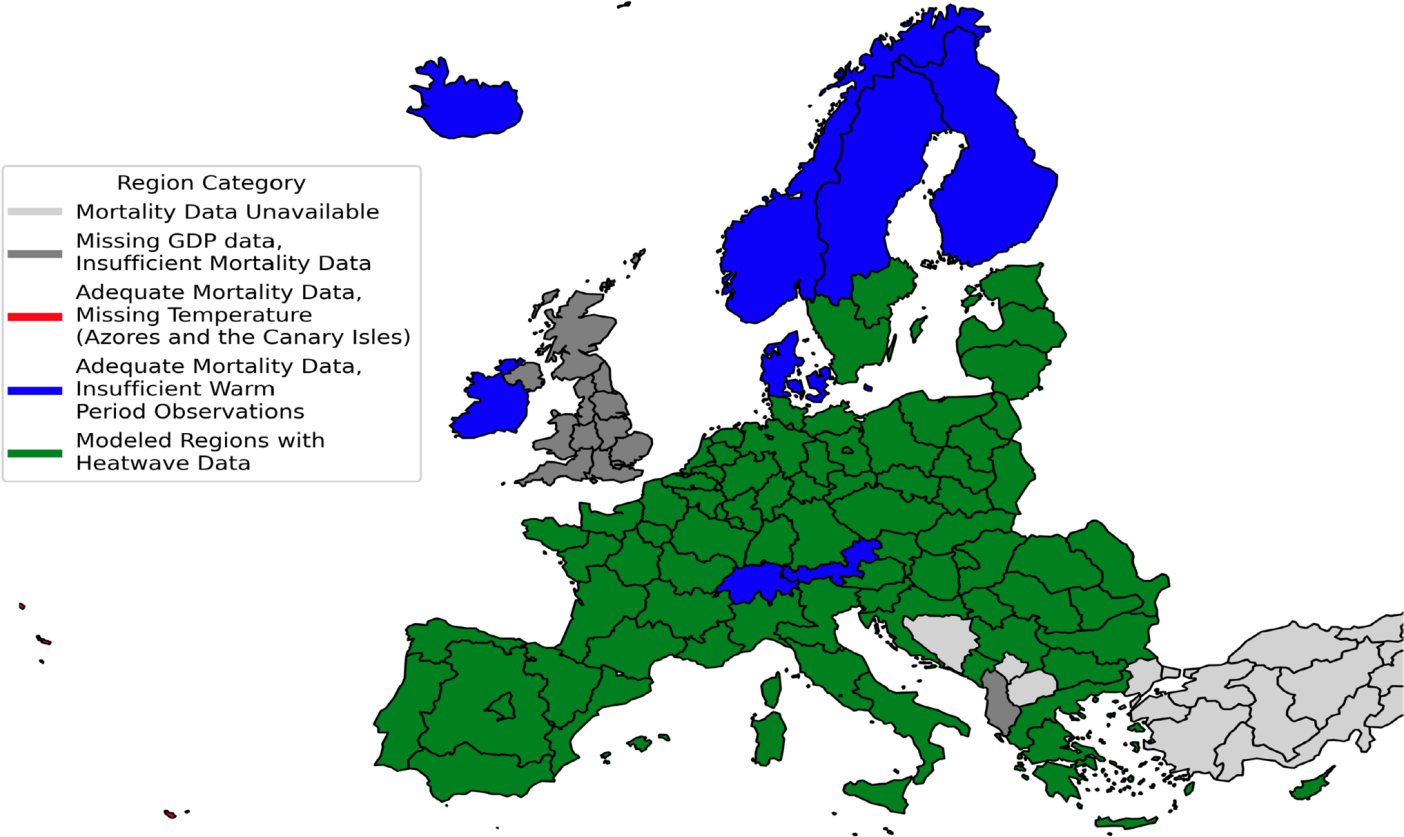
Regional data availability for heatwave impact modeling

**Fig. 2:**
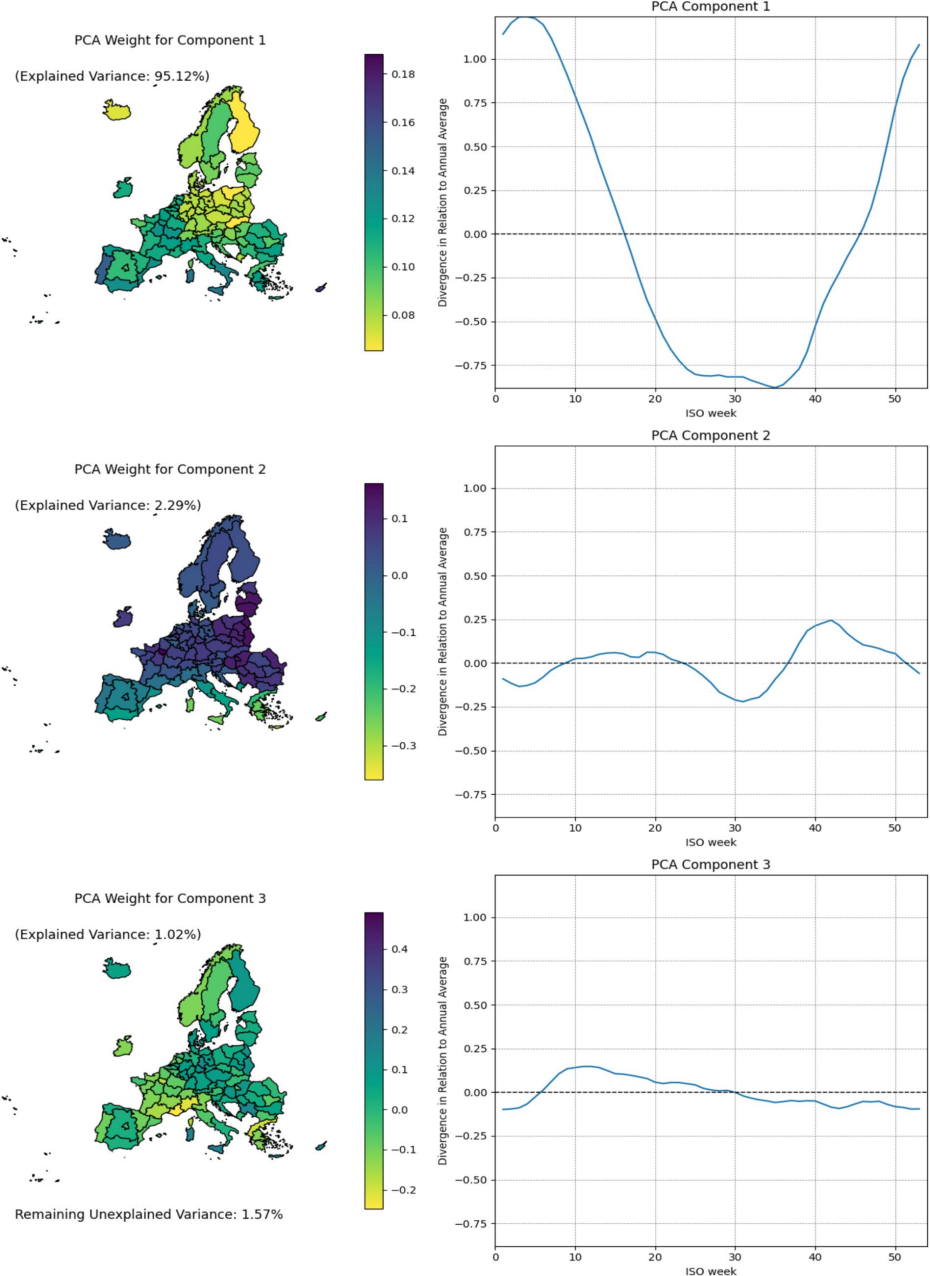
Underlying cycles extracted using PCA and their geographical distribution

Regional data availability is presented in Fig. 1, showing 94 NUTS1 regions with sufficiently long weekly death time series to model mortality baseline. The final analysis included 85 regions across 25 countries that had complete data across all required variables. The results of the PCA transformation of mortality baseline are presented in Fig. 2. The primary extracted dimension explains 95.12% of the variance and distinguishes between countries with long infection seasons and low annual amplitudes, and those with short but more intensive seasons (Douglas and Rawles 1999; Jones 2021; Madaniyazi et al. 2022). The second dimension, explaining 2.29% of the variance, reflects hot summers – the adjustment reflects both a much longer interruption of the infection season but with possibility of modest mortality increase in hottest period (Falagas et al. 2009; Walkowiak and Walkowiak 2024). The third dimension explains 1.02% of the variance and primarily serves as a time shift adjustment for the cycle. However, it also appears to capture more regular mortality peaks during the influenza season. 1.57%, primarily fitting noise, remained unexplained.

**Fig. 3:**
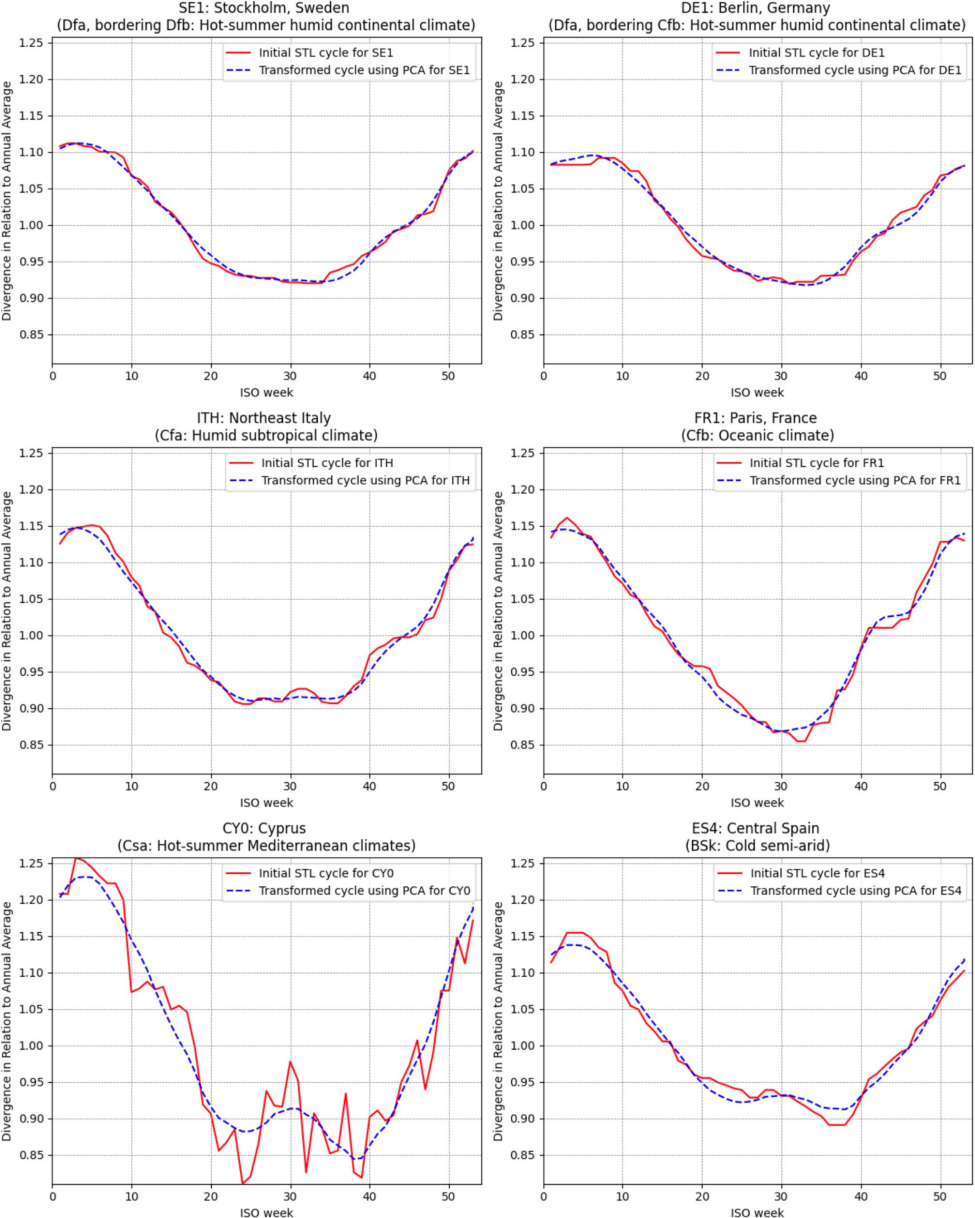
Comparison of trends extracted by the STL model and their form after PCA transformation

Figure 3 presents the results of modifying the initially calculated cycles using inverse transformation to obtain estimates in the original feature space for selected NUTS1 regions, with a descriptive designation of available climate zones within the sample. The resulting cycles are significantly smoother. Although the second PCA axis attributed some summer mortality increase to to the baseline, it was combined with primary axis that predicted a stronger midsummer dip. Thus, for Northeast Italy with Cfa (humid subtropical) or Central Spain with Bsk (cold semi-arid), this mostly canceled out. However, this caused some issues in the southernmost edges of Europe, like Cyprus with Csa (hot-summer Mediterranean), where a noticeable summer increase in mortality was attributed to the baseline. This issue couldn't be resolved without making highly arbitrary adjustments, so it was accepted. Importantly, as the model compared changes within regions, the impact mostly canceled out. Moreover, even in the hottest regions, the summer peak of the estimated baseline remained below the annual average and thus wasn't particularly adverse.

**Fig. 4:**
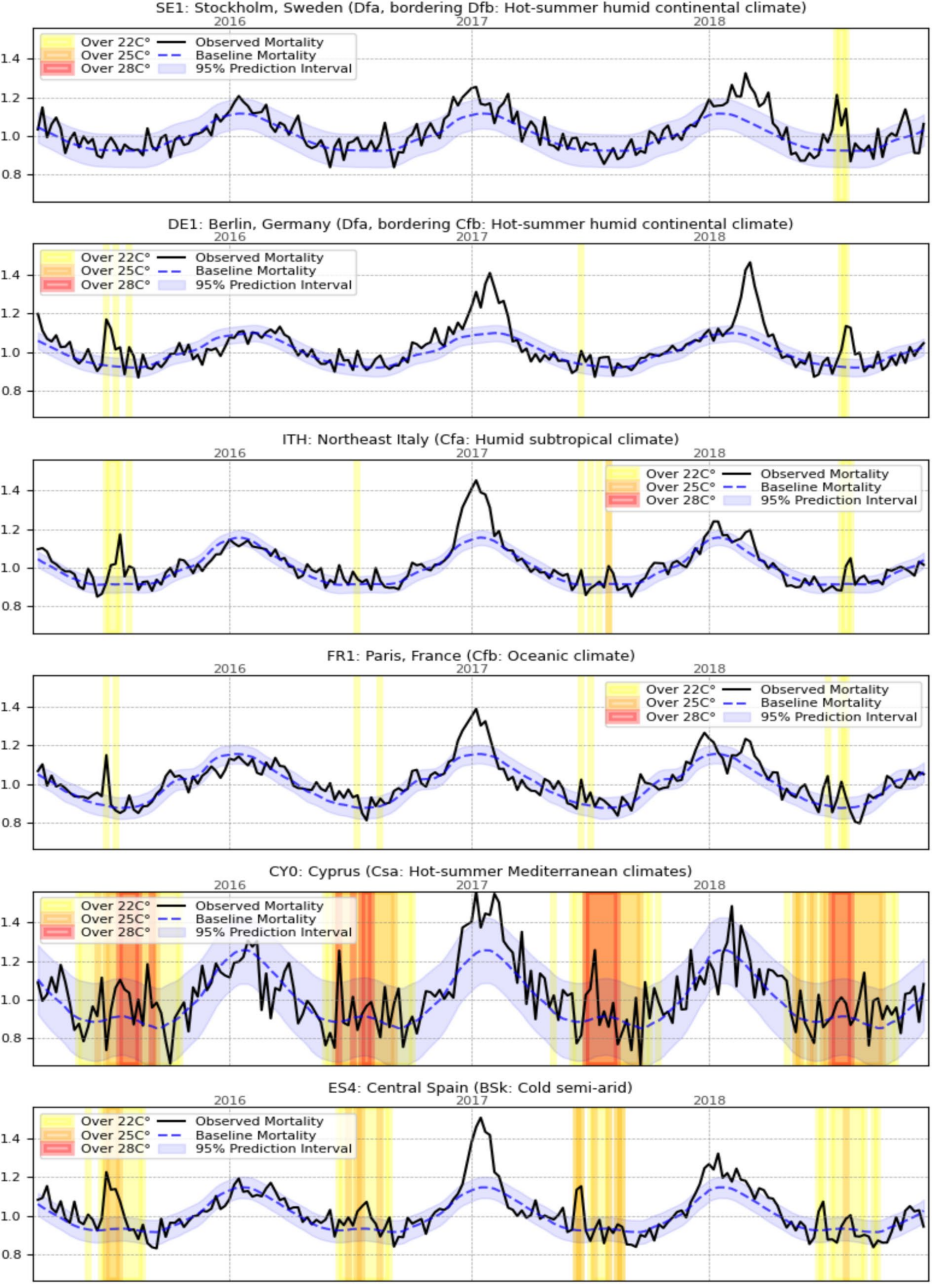
Example mortality fluctuations, baseline values, and periods of elevated temperature in selected regions

Figure 4 depicts an example time series presenting the complex relationship between temperature and mortality. Stockholm was selected as effectively the coldest region where there were a sufficient number of days for modeling the impact of heatwaves, and it was possible to demonstrate how exceeding a temperature of 22 °C led to a clear spike in heatwave mortality. This was especially noticeable as that region had a long infection season, thus much more flattened excess winter mortality. A similar pattern, albeit with more heatwave spikes, was noticeable for Berlin, which was at the edge of a continental climate. Nevertheless, while the same heatwave in midsummer 2018 also led to even higher temperatures in warmer regions, the increase in mortality there was much smaller. For Cyprus and Central Spain, it remained technically within the background noise level. Cyprus, while experiencing very high temperatures by European standards, nevertheless had relatively modest high temperature mortality and experienced much more elevated mortality in winter, especially during highly noticeable influenza waves in late winter.

Both Hausman test and pFtest obtained p-value below 0.001, what was a strong argument in favor of panel regression. Nevertheless the results were not noticeably sensitive to model selection, as coefficients of random effects model had overlapping confidence intervals while the only meaningful difference of linear regression was failing to detect statistically significant impact of economic output change. The panel models are outlined in Table 1 with all variables having statistical significance at level of p < 0.001. The initial model indicates that changes in resistance over time counteract the effect of a 1 °C increase every 17.9 years [95% CI 15.3–22.7]. However, introducing average summer temperature over the past three years and economic output renders the time variable statistically insignificant. Regardless of model employed, the best fit was achieved by considering only the past 3 years, with past climate lacking statistical significance even in linear regression. Consequently, to tolerate each additional 1 °C, per capita economic output must increase by 19.7 thousand euros [95% CI 14.6–30.3]. The addition of log-transformed economic output to represent diminishing marginal utility yields a variable lacking statistical significance, with a reversed sign, indicating the absence of diminishing returns.

**Table 1:** Panel regression predicting excess mortality in weeks with at least one day with a daily temperature of 22 °C

	Model 1		Model 2	
	Parameter	Std. Err	Parameter	Std. Err
Intercept	3.0300	0.2253	0.9679	0.0350
Year	−0.0012	0.0001		
Per capita GDP in euro in PPP			−1.085e-06	1.771e-07
90th percentile temperature from last 3 years			−0.0116	0.0015
Weekly temperature	0.0215	0.0004	0.0214	0.0004
Weekly temperature change	0.0017	0.0003	0.0019	0.0003
ISO week	−0.0019	0.0002	−0.0018	0.0002
Influenza deaths of preceding spring	−0.0055	0.0007	−0.0054	0.0007
R-squared (Overall):	14.21%		21.32%	
	No. Observations: 12,446		Entities: 85	

**Fig. 5:**
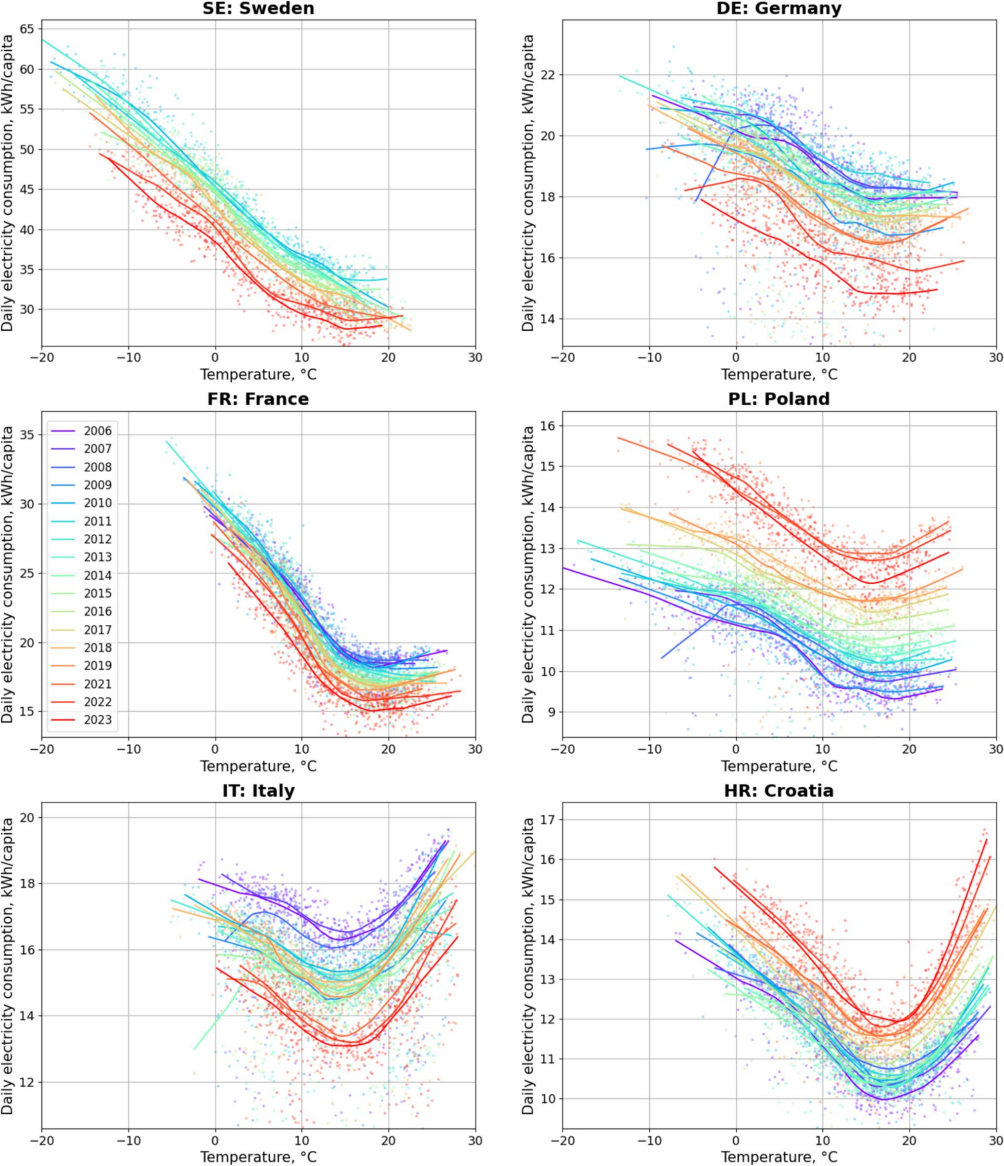
The Sensitivity of energy consumption to daily temperature in selected EU countries

The changes in energy demand sensitivity, presented in Fig. 5, reveal a nuanced picture. New EU members experiencing economic booms, such as Poland and Croatia, showed both an overall rise in energy consumption and heightened sensitivity of electricity use to rising temperatures. Conversely, old EU members exhibited decrease in overall energy consumption and no clear change in cooling energy demand. High temperature energy demand exceeds low temperature energy demand only in southeastern Europe. Even in France's oceanic climate, the summer increase was minimal. In the coldest continental countries, like Sweden, overall consumption was highest but actually decreased during the warmest periods.

## Discussion

During the analyzed period, Europe was gaining the capacity to tolerate an additional 1 °C rise every 17.9 years [95% CI 15.3–22.7]. Historical climate averages had weaker predictive value compared to contemporary patterns. Extending the temperature indicators beyond the prior 3 years did not enhance predictive accuracy, indicating swift adaptations to changing climate. Each additional 1 °C became tolerable with a per capita GDP increase of 19.7 thousand euros [95% CI 14.6–30.3], most likely due to greater air conditioning affordability. Energy usage shows increasing cooling energy demand, though still smaller heating energy demand.

The model's coefficients align with existing literature. While initial heatwaves lead to an increase in mortality, this impact diminishes in the following weeks due to the harvesting effect. Notably, even after adjusting for the natural decline in baseline mortality during summer, the model reveals a gradual increase in resistance to high temperatures over the season. This observation could be due to a delayed harvesting effect or might hint at rapid adaptation, a concept noted in performance sports literature (Tyler et al. 2016). Furthermore, spring epidemics appear to reduce the deadly impact of high temperatures (Stafoggia et al. 2009; Rocklöv et al. 2009; Ha et al. 2011; Qiao et al. 2015). The discovery of an optimal fit for a three-year lag is particularly intriguing; a shorter timeframe would likely be affected by random noise, implying rapid adaptation processes, which corroborates findings from a study focusing on a Spanish sample (Huber et al. 2022). The model predicts, as literature shows, that populations adapted to a specific temperature range are not fully immune but have developed high resistance, experiencing only modestly elevated mortality in adverse conditions (Gasparrini et al. 2015; Walkowiak et al. 2024). Thus, the faster increase in tolerance should be seen as outpacing the impact of climate change.

The model suggests that analyzed countries with relatively cold climates possessed significant capacity for rapid adaptation to warming temperatures, particularly when supported by economic development. While the possibility that modest infrastructural adaptations could fundamentally alter human vulnerability to temperature may seem counter-intuitive, such a process would not be unprecedented. Historically, before modern sanitation, moderately high temperatures were associated with peak annual mortality rates (Landers and Mouzas 1988; Alcoforado et al. 2015; Budnik and Liczbinska 2015). This explains why models assuming fixed vulnerability with no adaptation tend to diverge from subsequent observational data. Demonstrating the link between GDP and vulnerability to high temperatures could further improve the predictive value of models due to partial error cancellation when predicting economic pathways. Higher economic growth, though associated with higher emissions than anticipated, would also be related to lower vulnerability.

The lack of diminishing returns predicted by the model may seem somewhat unusual, as the Preston curve shows life expectancy rising with the logarithm of GDP (Mackenbach and Looman 2013; Jetter et al. 2019). This indirectly suggests that the mediating mechanism involves a good with income price elasticity above 1, known as a luxury good, where increased income leads to a disproportionately large increase in purchases. This pattern has been observed in energy demand for cooling (McNeil and Letschert 2010; Atalla et al. 2018). Other studies on air conditioning market penetration show a relationship between temperature and wealth, indicating that at low income levels, air conditioning is relatively rare, but starts growing rapidly from around $10,000 USD of household annual income (Davis et al. 2021). While this variable likely captures the combined impact of several infrastructural or behavioral adjustments, the results are closely aligned with air conditioning, which is further supported by the literature as a key adaptation strategy (Chua et al. 2023; Falchetta et al. 2024). While cooling energy demand remained mostly unchanged in the old EU countries, GDP growth was demonstrated to reduce heatwave mortality. Setting aside subtle efficiency gains, the most plausible explanation is that the market was mostly saturated, except that cooling solutions finally became available for the most frail and vulnerable.

The observed relationship has clear policy implications. Recent landmark reports emphasize the importance of green spaces and public health interventions (Daalen et al. 2022, European Environment Agency 2024), while viewing air conditioning with worrisome ambivalence. While they acknowledge that wealthier households are less vulnerable to heatwaves and the need to address energy poverty, the emphasis lies on overhauling the energy infrastructure, which is a costly and potentially conflicting goal. On one hand, objections suggest that it may undermine decarbonization efforts and the introduction of less energy-intensive cooling solutions (Daalen et al. 2022), which while advantageous for new buildings have limited economic viability in case retrofitting existing buildings (Oropeza-Perez and Østergaard 2018; Sun et al. 2018). On the other hand, concerns relate to its potential to compound stress on an energy grid already experiencing challenges in cooling thermal power plants (European Environment Agency 2024).

The objections regarding the increase in emission and the strain on the energy grid, while seemingly valid as the warmest EU countries indeed show moderately higher wholesale electricity prices in summer than in winter (Bigerna 2018), do not account for the ongoing energy transition. This transition reduces the role of thermal power plants and increases renewable energy penetration, which, due to its intermittency, must be supported by increased interconnectedness, spare capacity, and storage (Carlini et al. 2019; Tan et al. 2023). Recent research suggests that planning inadequately addresses the risks of extreme winter weather, with polar vortex events in the US resulting in record-breaking energy prices serving as a prime example (Michelfelder and Pilotte 2021). Numerous factors are likely to escalate strain during the winter. Studies suggest that the grid's primary stress point will be the Dunkelflaute, a frequent high-pressure, low-temperature system during winter characterized by low solar and wind production and increased energy demand (van der Wiel et al. 2019; Li et al. 2021). The EU policy of reducing reliance on fossil fuel heating by promoting heat pump installations (European Comission 2024) has important implications for energy demand. Empirical studies on heat pump adoption have generally concluded that their installation leads to a significant increase in heating demand, while cooling demand remains effectively unchanged (Liang et al. 2022) or may even decrease slightly (White et al. 2021). In the rare cases where heat pumps replace instead direct electric heating overall energy efficiency improves dramatically. However, due to the declining efficiency of air-source heat pumps in very low temperatures and the economic rationale of sizing them for typical demand (Dongellini et al. 2021), in case of cold snaps they require back up, typically either gas boiler or direct electric heating (Waite and Modi 2020). Thus, policies are likely to increase grid sensitivity to low temperatures even more than illustrated in Fig. 4, with UK estimates suggesting a 20% increase in peak winter demand in the next 15 years (Staffell and Pfenninger 2018), compared to the single-digit energy demand change predicted for the most climate-sensitive EU regions due to climate change by the end of the century (Wenz et al. 2017). To keep renewable power curtailment low (Yasuda et al. 2022), increasing interconnectedness should make countries less vulnerable to local shocks. Moreover, fluctuating risk premiums suggest that transfer issues are most severe in winter (ACER 2023). Therefore, since the risk of summer energy shortages appears exaggerated, the focus should shift to promoting economic development or implementing targeted policies to address energy poverty among the most vulnerable groups.

The observed adaptations cannot be explained by favorable policies alone, as selected policies, although beneficial in other ways, appear to rather impede adaptation to this specific climate threat. Despite attempts to mitigate rising renewable energy costs for consumers (Trujillo-Baute et al. 2018), the impact remains significant, leading even to decreasing public support (Aklin 2021). Furthermore, policies promoting improved insulation inadvertently escalate summer cooling energy demands (Mlakar and Štrancar 2011; Willand et al. 2016).

### Limitations

The best fit observed after including the last three years of temperature data should be understood as the period after which the modest impact of further adaptations is likely overshadowed by subsequent temperature changes. While the link between economic development, energy usage, and resistance to high temperatures may seem straightforward, it likely oversimplifies the relationship and the specific adaptations required for different temperature ranges and changes in age structure. The presented graphs depicting energy consumption likely underestimate the change by disregarding efficiency gains. The need for reserve capacity to avoid blackouts during Dunkelflaute is a local phenomenon and does not apply in warmer regions.

While model estimates are consistent with prior studies documenting the paradoxical decrease in high temperature-mortality despite a warming climate and appear as much more plausible assumption than fixed relationship, caution in extrapolation is needed. The sample primarily included regions located in continental and temperate climates, which were, at the onset of the analyzed period, at least of lower medium-income (World Bank 2024). Our study focused on European, relatively cold countries where adaptation to warming may be more achievable, thus physiological limits to human adaptation had not been detectable in that range. They would be likely a limiting factor in case of much warmer climates, though decreases in heat-related mortality despite warming have been observed even in humid subtropical climates like Hong Kong (Chau and Woo 2015). Particularly risky may be the extrapolation of the linear role of GDP through air conditioning as a mediatory variable, as it is unlikely to hold true for the poorest countries, as prior studies show a minimum income level (Davis et al. 2021), nor for the richest countries, which could achieve market saturation leading to diminishing returns. Moreover, decade-long studies show that the observed mortality-temperature relationship clearly undergoes long-term shifts (Carson et al. 2004; Alcoforado et al. 2015), and extrapolating contemporary relationships into the past would miss historical observational data. Therefore, we suggest extra caution in extrapolating contemporary relationships beyond at most a few decades, even after including adjustments suggested by us.

The model only predicts the impact of high temperatures on human mortality and neither accounts for damage to the overall ecosystem nor changes in mortality due to other natural disasters (Visser et al. 2014; Paprotny et al. 2018). The sole pathway through which the model can consider indirect damage to the overall ecosystem is when it becomes severe enough to have a meaningful economic impact, for example, due to damage to agriculture (Moriondo et al. 2010) or tourism (Damm et al. 2017). Additionally, while the model adjusts for the impact of recurring influenza epidemics that take the lives of the most vulnerable in already early spring, it is calibrated for a particular epidemic timing and virality range. Thus, a sudden change in the dominant strain, like the one observed in 1968 caused by antigenic shift (Jones and Ponomarenko 2022; Brüssow 2022), could alter this coefficient. Excluding the COVID-19 pandemic from the model slightly reduces its performance. The estimated impact of an early-year epidemic shifts from −0.0054 [95% CI: −0.0067, −0.0041] to −0.0073 [95% CI: −0.0093, −0.0052], representing the only notable change among variables. However, the overlapping confidence intervals highlight the uncertainty of this adjustment.

Due to the weekly resolution of our data, the model cannot detect temporal displacement of deaths within the same week. While this consistent measurement approach throughout the analyzed period preserves our ability to detect directional changes, comparisons with daily-data studies suggest that weekly estimates may be lower by approximately 21.56%, primarily for moderate heat exposure (Ballester et al. 2024). Nevertheless it remains an open methodological question whether that short-term mortality displacement maintain clinical significance to be counted as additional heat-related deaths.

## Data Availability

All data used is publicly available.
